# Heterogeneity in susceptibility to polycystic ovary syndrome among women with epilepsy

**DOI:** 10.1186/s42494-023-00125-4

**Published:** 2023-06-19

**Authors:** Leihao Sha, Yiming Wu, Wanlin Lai, Yifei Duan, Yilin Xia, Lei Chen

**Affiliations:** Department of Neurology, Joint Research Institution of Altitude Health, West China Hospital, Sichuan University, No. 37 Guo Xue Xiang, Chengdu, Sichuan 610041 PR China

**Keywords:** Epilepsy, Polycystic ovary syndrome, Gene, Impaired metabolism

## Abstract

**Background:**

Epilepsy comorbidities adversely affect the quality of life of patients. Women with epilepsy are at a high risk of comorbid endocrine disorders. Among them, the polycystic ovary syndrome (PCOS) has a threefold higher prevalence in women with epilepsy than in healthy women and is the main cause of infertility among the patients. Clinically, women with epilepsy show heterogeneity in the susceptibility to PCOS. This heterogeneity may be associated with genetic factor.

**Methods:**

To test this, we retrospectively collected clinical data from 45 female patients with epilepsy and divided them into three groups according to their susceptibility to PCOS. Groups A and B represented a high susceptibility to PCOS. Patients in Group A were diagnosed with PCOS before their first seizure, while patients in Group B were diagnosed with PCOS after a short period of monotherapy with a low dose of antiseizure medication (ASM) following the diagnosis of epilepsy. Patients in Group C did not develop PCOS despite a prolonged treatment with high-dose ASM. We compared the clinical data and genetic profiles among the three groups.

**Results:**

We found a clear trend of impaired metabolism in Group B patients and this may be associated with high-frequency mutations in *MYO10* and *ADGRL3*.

**Conclusions:**

Our study suggests that women with epilepsy are heterogeneous in the susceptibility to PCOS and this is associated with mutations in specific genes. Therefore, genetic screening should be conducted to screen for women with epilepsy who are more likely to have comorbid PCOS, so that they can receive targeted interventions at an early stage to reduce the risk.

**Supplementary Information:**

The online version contains supplementary material available at 10.1186/s42494-023-00125-4.

## Background

Epilepsy is one of the most common chronic neurological diseases, affecting 69 million individuals globally and negatively affects the health and quality of life of patients [1]. In addition to unpredictable seizures, epilepsy comorbidities have a huge impact on patient’s quality of life, and almost all people with epilepsy have one or more comorbidities [2, 3], causing a significant disease burden for society [4]. Among them, comorbidities related with reproductive endocrine have a serious impact on the full cycle of health management in women with epilepsy [5, 6]. Women have a more complex endocrine system and show marked cyclical fluctuations. As a result, women with epilepsy are prone to reproductive endocrine disorders. Several studies have reported decreased blood levels of estradiol and progesterone and increased prolactin levels in women with epilepsy [7–9]. In addition, testosterone levels in epileptic women are frequently elevated, resulting in hyperandrogenism symptoms and the polycystic ovary syndrome (PCOS). It is estimated that more than 15-30% of women with epilepsy have PCOS, higher than the 10-20% in the normal population [10–12]. PCOS is a major cause of infertility in women with epilepsy [13]. In addition, long-term disruption of the endocrine system can lead to abnormal glucose tolerance, complicating insulin resistance and even diabetes [14]. Previous studies suggest that both inappropriate antiseizure medication (ASM) use and uncontrolled seizures can induce reproductive endocrine disorders in women with epilepsy [15] and that the use of valproic acid (VPA) use in particular is highly associated with the development of PCOS. However, some recent studies in large samples have shown that VPA is not associated with a high incidence of PCOS in women with epilepsy. Bauer et al. reported that 35.5% of female patients with focal epilepsy (*n* = 93) had PCOS and this was not associated with VPA use [16]. This finding is consistent with earlier report by Luef et ak. and Zhou et al. [10, 12]. Therefore, there may be other factors involved in the comorbidity of PCOS in women with epilepsy than VPA use.

In clinical practice, our team has also found that some women with epilepsy are extremely susceptible to PCOS, exhibiting two types of susceptibility. The first is a high susceptibility to PCOS before the onset of epilepsy and without receiving any anti-epileptic drug treatment. The second is a sensitivity to adverse drug reactions, where PCOS occurs within a short time of a small-dose ASM monotherapy. The heterogeneity in susceptibility to PCOS in these patients may represent two different phenotypes of susceptibility to the epileptic co-morbid PCOS. Whether this difference in susceptibility is accompanied by a difference in clinical features remains unknown. In addition, this heterogeneity in susceptibility may also be associated with genetic factors. Previous studies have reported separate susceptibility genes for epilepsy and PCOS, but whether these genes are involved in the association between epilepsy and PCOS remains unclear [17–19]. If the PCOS susceptibility could be accurately predicted in women with epilepsy based on genetic screening, precise interventions on lifestyle and treatment can be provided to reduce the incidence of PCOS and improve the quality of life of women with epilepsy. Based on the above evidence, we propose that there is a heterogeneity in the genetic factor-associated susceptibility to epilepsy co-morbid PCOS. In this study, we retrospectively analysed the clinical characteristics and genotypes of women with epilepsy with different susceptibilities to PCOS, and compared them with those patients without PCOS.

## Methods

### Data collection and study population

In this retrospective case–control study, Chinese patients with diagnosis of epilepsy were recruited in West China Hospital, Sichuan University from July, 2017 to July, 2022. The inclusion criteria were female sex, age ≥ 14 years, not entering menopause, and with available laboratory data (sex hormones, fasting plasma glucose, oral glucose tolerance test, serum insulin) and ultrasound of ovary. Females aged 14–16 experienced menarche at least two years ago. The type and etiology of epilepsy were classified according to the 2017 ILAE classification. Patients were classified into three groups based on the diagnosis of PCOS by a professional gynecologist according to the 2003 Rotterdam diagnostic criteria. Group A included patients with PCOS diagnosed before epilepsy. Group B included patients diagnosed with PCOS within 5 years of low-dose ASM monotherapy. Group C included patients without PCOS after high-dose ASM therapy for more than 5 years. For detailed criteria of each group, see Supplementary Material 1.

### Clinical data collection

All relevant clinical data were extracted from electronic medical records via the Clinical Research and Exploration System of West China Hospital, Sichuan University. All participants included were informed about the study and signed a written informed consent form. This study was approved by the West China Hospital of Sichuan University Biomedical Research Ethics Committee. The following data were collected for each patient: age, sex, body mass index (BMI), age of first seizure onset, ASM treatment history, fasting plasma glucose, oral glucose tolerance test [GLU], serum insulin [INS], sex hormones (including dehydroepiandrosterone sulfate [DHEA-S], luteinizing hormone [LH], follicle stimulating hormone, prolactin, progestin, estradiol and testosterone) and ultrasound of the ovaries. Sex hormones and ultrasound were examined 2–3 days after the first day of menstruation. Additionally, we calculated the Homeostatic Model Assessment of Insulin Resistance (HOMA-IR) by multiplying fasting plasma glucose with fasting insulin. HOMA-IR ≥ 60 indicates insulin resistance. We also assessed fasting insulin (FINS) in all patients. If the fasting plasma insulin was > 10 μU/ml, the patient was considered with insulin resistance. Patients with BMI ≥ 24.0 were considered as overweight.

### Gene data collection

All patients included were asked to participate in gene analysis willingly. And blood samples of patients who agreed to participate in gene analysis were obtained for DNA sequencing. We extracted all genes reported to be related to epilepsy and PCOS from databases ClinVar (V2022-10-1), the Human Gene Mutation Database (HGMD) (see HGMD in web resources) and GWAS Catalog. ClinVar provided variants found in diseased patients with supporting evidence. HGMD included mutations collected from published works. The GWAS Catalog archived SNP-trait associations from 5848 publications of GWAS (release of 2022-7-9). "Polycystic_ovary_syndrome" and "Epilepsy" were used as keywords to screen for mutations in ClinVar and HGMD. Variants labeled with "Pathogenic" in ClinVar were retained, while the "DM" and "DM?" variants were included from HGMD. In the GWAS Catalog, we collected all variants from curated GWAS results for PCOS and EP. Finally, a total of 5563 epilepsy-related and 158 PCOS-related variants were included, of which 243 epilepsy-related and 50 PCOS-related variants were covered by our sequencing samples, respectively. The results of DNA sequencing were interpreted in comparison to reported genes related to epilepsy and PCOS. Because of the limited sample size, we did not perform any test to the results, but instead present top ten gene variants ranked in the order of allele frequency in each group.

### Statistical analysis

Data are presented as mean ± standard deviation (SD) for continuous variables and as frequencies for categorical variables. Analysis of variance was performed to compare continuous variables between groups. We did not perform any test to compare categorical variables between groups regarding the rather small sample in this study. The allele frequencies of variants were calculated for each group. All analyses were conducted using R (Version 4.0.4) and Plink (Version 1.90) softwares. A two-sided *P* value < 0.05 was considered as statistically significant.

## Results

A total of 45 patients were included in this study (*n* = 10 in Group A, 10 in Group B and 25 in Group C), of whom 28 patients agreed for DNA sequencing (*n* = 7 in Group A, 5 in Group B and 16 in Group C). Table 1 shows the clinical characteristics of the participants by group. Patients in Group C were significantly older than those in other groups (*P* = 0.005), because they had a long history of ASM treatment. There was no significant difference in the ASM treatment history among the three groups. Levetiracetam was the most used ASM in all three groups. The Group B showed a higher portion of impaired glucose metabolism characterized with insulin resistance (HOMA-IR: Group A 10%, Group B 50%, and Group C 25%; FINS: Group A 30%, Group B 60%, and Group C 24%). The Group B also showed a significantly higher plasma glucose level at 2 h in oral glucose tolerance test than Group A, but did not differ from Group C. The Group B showed a higher frequency of overweight (Group A 10%, Group B 50%, and Group C 28%). For sex hormones, Groups A and B showed significant increases of DHEA-S, LH, and testosterone compared to Group C.

**Table 1:** Clinical characteristics of three groups

	Group A (*n* = 10)	Group B (*n* = 10)	Group C (*n* = 25)	*P* value
Age (years)	22.2 ± 3.3	20.5 ± 3.7	28.12 ± 7.5	0.005^*^
Onset (years)	19.8 ± 2.4	10.5 ± 4.1	15.72 ± 8.3	0.354
ASM				
Beginning age (years)	20.3 ± 3.1	16.7 ± 5.2	17.5 ± 8.5	0.365
VPA, *n* (%)	0	1 (10%)	9 (36%)	
TPM,* n* (%)	0	1 (10%)	3 (12%)	
CBZ,* n* (%)	0	0	12 (48%)	
LEV,* n* (%)	7 (70%)	4 (40%)	11 (44%)	
OXC,* n* (%)	1 (10%)	2 (20%)	9 (36%)	
LTG,* n* (%)	1 (10%)	3 (30%)	7 (28%)	
PB,* n* (%)	0	0	3 (12%)	
LH	11.48 ± 9.53	9.81 ± 5.48	6.65 ± 3.72	0.022^*^
FSH	6.08 ± 1.20	6.08 ± 1.57	6.81 ± 3.24	0.396
LH/FSH	1.86 ± 1.37	1.56 ± 0.58	1.24 ± 1.20	0.135
PRL	17.27 ± 8.04	21.74 ± 5.67	34.17 ± 45.9	0.167
E2	54.66 ± 31.12	37.41 ± 9.89	67.25 ± 83.46	0.456
P	0.41 ± 0.28	0.37 ± 0.27	0.88 ± 1.76	0.277
T	0.50 ± 0.09	0.36 ± 0.17	0.21 ± 0.11	< 0.001^*^
DHEA-S	8.35 ± 2.79	7.45 ± 3.45	3.76 ± 1.93	< 0.001^*^
BMI	20.9 ± 1.7	24.7 ± 6.7	21.9 ± 3.0	0.822
Overweight, *n* (%)	1(10%)	5(50%)	7(28%)	
GLU-0	4.85 ± 0.30	5.23 ± 0.41	5.03 ± 0.39	0.413
GLU-2 h	5.63 ± 1.11	6.81 ± 2.99	7.24 ± 1.78	0.041^*^
INS-0	8.33 ± 2.01	19.13 ± 19.89	8.61 ± 3.55	0.589
INS-2 h	50.72 ± 19.95	114.19 ± 135.55	58.75 ± 33.30	0.844
FINS, *n* (%)	3(30%)	6(60%)	6(24%)	
HOMA-IR, *n* (%)	1(10%)	5(50%)	5(25%)	

Onset: age of first seizure; *ASM* antiseizure medication, *VPA* valproic acid, *TPM* topiramate, *CBZ* carbamazepine, *LEV* levetiracetam, *OXC* oxcarbazepine, *LTG* lamotrigine, *PB* phenobarbital, *LH* luteinizing hormone, *FSH* follicle stimulating hormone, *PRL* prolactin, *E2* estradiol, *P* progestin, *T* testosterone, *DHEA-S* dehydroepiandrosterone sulfate, *BMI* body mass index, *GLU-0* fasting plasma glucose, *GLU-2 h* plasma glucose at 2 h in oral glucose tolerance test, *INS-0* fasting serum insulin, *INS-2 h* serum insulin at 2 h in oral glucose tolerance test, *FINS* fasting insulin test, *HOMA-IR* Homeostatic Model Assessment of Insulin Resistance

^*^P < 0.05

For gene analysis, the top 10 gene variants sorted by allele frequency in each group are listed in Table 2. The epilepsy-associated genes *KLRC4* and *NEMCE2* were high-frequency variants in all three groups. *PCDH7* and *KRTAP8-1* were high-frequency variants in epilepsy patients with PCOS, which could be associated with the susceptibility to PCOS in epilepsy patients.

**Table 2:** Top 10 variants in each group, sorted by allele frequency

Group	SNP	Gene	Full name	Gene ID	Gene type	Allele frequency
**Group A**	chr11:102,724,404:T:C	*MMP8*	Matrix metallopeptidase 8	4317	Protein-coding	0.5714
	chr12:10,408,358:T:C	*KLRC4*	Killer cell lectin like receptor C4	8302	Protein-coding	0.5714
	chr8:9,811,825:T:C	*-*	-	-	-	0.5714
	chr6:31,372,381:A:G	*-*	-	-	-	0.5
	chr4:31,149,735:G:A	*PCDH7*	Protocadherin 7	5099	Protein-coding	0.5
	chr21:30,811,678:G:A	*KRTAP8-1*	Keratin associated protein 8–1	337,879	Protein-coding	0.5
	chr1:67,287,825:C:T	*-*	-	-	-	0.5
	chr8:125,334,735:G:A	*NSMCE2*	NSE2 (MMS21) homolog, SMC5-SMC6 complex SUMO ligase	286,053	Protein-coding	0.5
	chr2:62,325,541:A:G	*-*	-	-	-	0.5
	chr11:46,777,081:A:G	*CKAP5*	Cytoskeleton associated protein 5	9793	Protein-coding	0.5
**Group B**	chr21:30,811,678:G:A	*KRTAP8-1*	Keratin associated protein 8–1	337,879	Protein-coding	0.7
	chr12:10,408,358:T:C	*KLRC4*	Killer cell lectin like receptor C4	8302	Protein-coding	0.7
	chr8:125,334,735:G:A	*NSMCE2*	NSE2 (MMS21) homolog, SMC5-SMC6 complex SUMO ligase	286,053	Protein-coding	0.7
	chr1:67,287,825:C:T	*-*	-	-	-	0.6
	chr4:46,238,270:G:T	*-*	-	-	-	0.6
	chr5:16,835,896:T:C	*MYO10*	Myosin X	4651	Protein-coding	0.6
	chr4:61,587,491:A:G	*ADGRL3*	Adhesion G protein-coupled receptor L3	23,284	Protein-coding	0.5
	chr6:31,368,641:G:A	*-*	-	-	-	0.5
	chr6:31,372,381:A:G	*-*	-	-	-	0.5
	chr4:31,149,735:G:A	*PCDH7*	Protocadherin 7	5099	Protein-coding	0.5
**Group C**	chr12:10,408,358:T:C	*KLRC4*	Killer cell lectin like receptor C4	8302	Protein-coding	0.5938
	chr12:112,825,713:A:C	*RPH3A*	Rabphilin 3A	22,895	Protein-coding	0.5625
	chr5:66,856,430:A:G	*MAST4*	Microtubule-associated serine/threonine kinase family member 4	375,449	Protein-coding	0.5625
	chr2:62,325,541:A:G	*-*	-	-	-	0.5312
	chr11:98,216,871:C:T	*-*	-	-	-	0.5
	chr8:9,811,825:T:C	*-*	-	-	-	0.4688
	chr16:79,721,183:C:A	*LINC01229*	Long intergenic non-protein coding RNA 1229	101,928,248	ncRNA	0.4688
	chr8:125,334,735:G:A	*NSMCE2*	NSE2 (MMS21) homolog, SMC5-SMC6 complex SUMO ligase	286,053	Protein-coding	0.4375
	chr1:67,287,825:C:T	*-*	-	-	-	0.4375
	chr4:46,238,270:G:T	*-*	-	-	-	0.4375

## Discussion

In this study, we reported for the first time the differences in clinical features and genetic profiles between epilepsy patients with and without PCOS. Both PCOS-susceptible groups had abnormal sex hormone levels compared to the non-susceptible group, which is consistent with the clinical features of PCOS. In addition, we compared the characteristics of patients presenting with PCOS before the onset of epilepsy (Group A) with the characteristics of those presenting with PCOS after a short period of ASM treatment after the onset of epilepsy (Group B). We found that patients who developed PCOS after a short period of ASM treatment after the onset of epilepsy tended to have more severe metabolic impairment, mainly in the form of insulin resistance and overweight. This may reveal the differences in PCOS phenotypes in women with epilepsy. Our study suggests that epilepsy patients who are sensitive to adverse reactions to ASMs are more likely to have impaired metabolism. For genetic profiles, different high-frequency mutations were observed in each of the three groups, which may be responsible for the different phenotypes that emerged.

Previous studies have suggested that VPA use is an important factor in the development of PCOS in patients; however, only one of the 20 epilepsy patients with PCOS received VPA treatment, suggesting that the VPA-related adverse effects are not a confounding factor in this study. Dapas et al. provided a comprehensive summary of genetic research on PCOS, suggesting that PCOS can be divided into three subtypes, reproductively impaired, metabolically impaired and intermediate [19]. Insulin resistance, hyperinsulinism and obesity are features of the metabolically impaired type. Our study suggests that although all of the epilepsy patients with co-morbid PCOS received ASM treatment, the PCOS that developed after a short period of ASM treatment (Group B) was more likely to be the metabolically impaired type, whereas this feature was not present in patients who developed PCOS before the onset of epilepsy. Therefore, clinicians should make more appropriate recommendations for the treatment of such patients who are sensitive to adverse reactions of ASMs and regularly test the severity of their metabolic impairment.

In addition, we found that of the six genes with high-frequency mutations, *NSMCE2* [20], *MYO10* [21], *ADGRL3* [22], and *PCDH7* [23] were all associated with abnormal glucose metabolism or insulin resistance, which is consistent with the unique PCOS phenotype of the Group B. Among them, *NSMCE2* and *PCDH7* also showed high-frequency mutations in Group A. These two genes may not be associated with patients' susceptibility to adverse effects of ASMs. Only Group B exhibited high-frequency mutations for *MYO10* and *ADGRL3*, which may explain the distinct PCOS phenotype of this group. *MYO10* has been reported to be associated with axon outgrowth and cell-to-cell communication in cortical neurons [24, 25]. *ADGRL3* has also been reported to be associated with neuronal migration and synaptic function [26]. Combined with their potential role in glucose metabolism and insulin resistance, such evidence suggests that *MYO10* and *ADGRL3* could be related to the underlying mechanisms of the comorbidity of PCOS in epilepsy. Studies are needed to further clarify their roles.

**Fig. 1:**
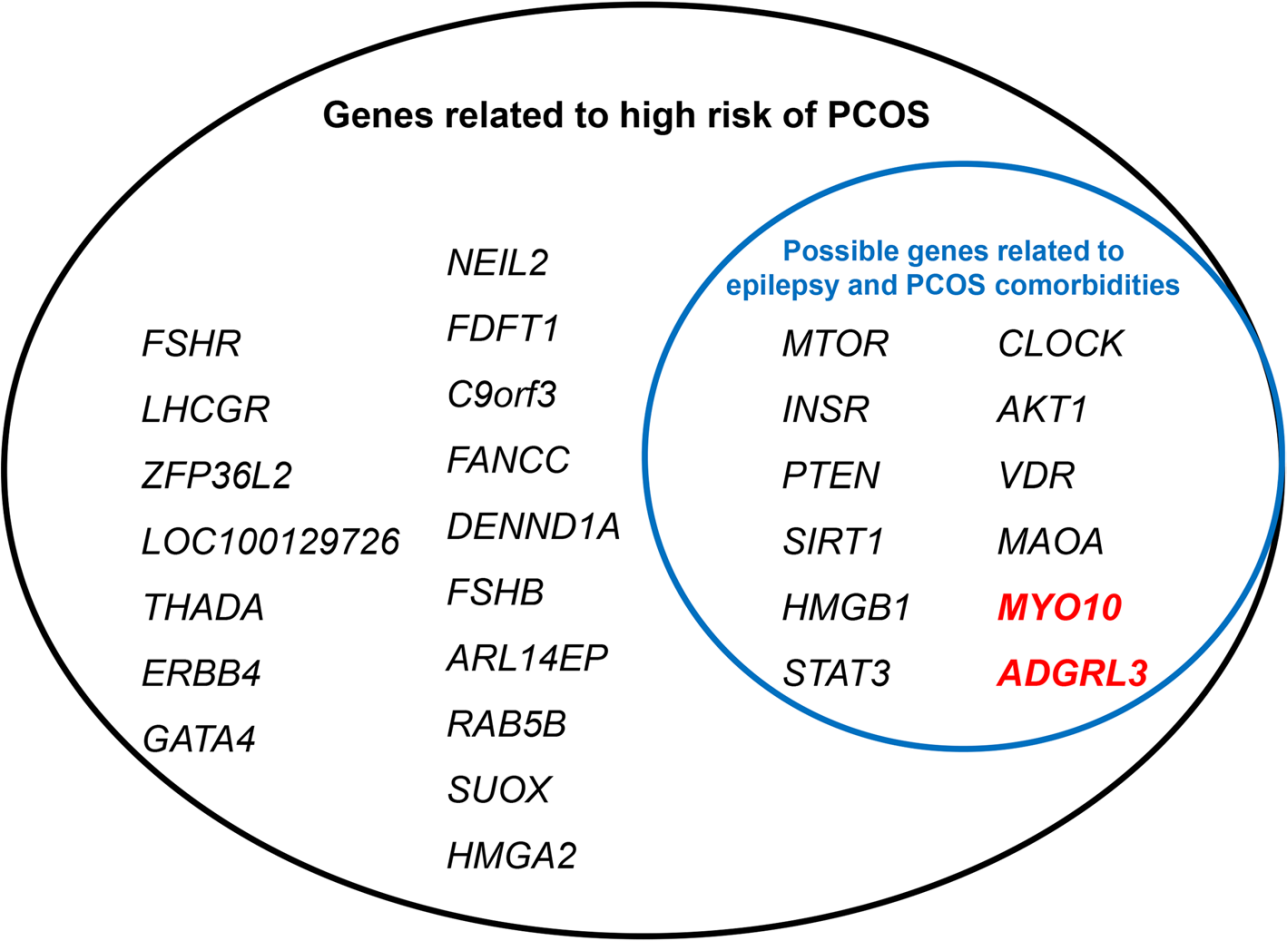
Classical genes related to high risk of PCOS and possible genes related to the comorbidity of PCOS in epilepsy [27–46]

We further summarized PCOS-related genes and epilepsy-related genes by literature search [27]. Classical genes related to the high risk of PCOS and possible genes related to the comorbidity of epilepsy and PCOS are demonstrated in Fig. 1. These genes could provide directions for future studies on the genetic relationship between PCOS and epilepsy and the early screening of PCOS in women with epilepsy.

This study has some limitations. The main limitation is the relatively small sample size, which limits the credibility of results. The statistical validity of small-sample studies is insufficient, so we used descriptive statistics for most of the data and only calculated *P* values for key data. From the limited data, we identified for the first time heterogeneous clinical presentations in epilepsy patients with PCOS. The heterogeneity needs to be further confirmed by studies with larger sample sizes.

## Conclusions

There are heterogeneous susceptibilities to PCOS among patients with epilepsy. Patients diagnosed with PCOS after a short period of low-dose ASM monotherapy are more likely to have impaired metabolism, especially insulin resistance and overweight. This group of patients also have high-frequency mutations of *MYO10* and *ADGRL3*, which may facilitate precise early screening of PCOS in female patients with epilepsy.

## Supplementary Information

**Supplementary Material 1.**

## Data Availability

The data that support the findings of this study are available from the corresponding author upon reasonable request.

## Abbreviations

ASM: Antiseizure medication
BMI: Body mass index
DHEA-S: Dehydroepiandrosterone sulfate
FINS: Fasting insulin test
GLU-0: Fasting plasma glucose
GLU-2 h: Plasma glucose at 2 h in oral glucose tolerance test
HOMA-IR: Homeostatic Model Assessment of Insulin Resistance
INS-0: Fasting serum insulin
INS-2 h: Serum insulin at 2 h in oral glucose tolerance test
LH: Luteinizing hormone
PCOS: Polycystic ovary syndrome
VPA: Valproic acid
