# Supplementary material for: Heterogeneity in susceptibility to polycystic ovary syndrome among women with epilepsy
Source: Acta Epileptol. 2023 Jun 19;5:14. doi: 10.1186/s42494-023-00125-4 (PMC11960223; doi:10.1186/s42494-023-00125-4)
Supplement: Supplementary file 1 — Supplementary Material 1. [file 42494_2023_125_MOESM1_ESM.docx]

**Detailed criteria of each group:**

**Group A:** diagnosed with PCOS before their first seizure.

**Group B:** diagnosed with PCOS after a short period of monotherapy with a low dose of antiepileptic drugs following the diagnosis of epilepsy. With a literature search of previous studies on the comorbidity of epilepsy and PCOS, we defined the included criteria based on the reported antiepileptic drugs and their doses. A short period refers to less than 5 years of antiepileptic drug monotherapy. A low dose of antiepileptic drugs refers to one of the following situations: valproic acid sodium less than 800mg per day, lamotrigine less than 150mg per day, carbamazepine less than 500mg per day, oxcarbazepine less than 1000mg per day, or phenytoin less than 250mg per day.

**Group C:** diagnosed with epilepsy only with complete PCOS screening after prolonged high-dose antiepileptic drug treatment. These patients also have recorded seizures with a frequency of more than once a month. With a literature search of previous studies on the comorbidity of epilepsy and PCOS, we defined the included criteria based on the reported antiepileptic drugs and their doses. A prolonged period refers to more than 5 years of antiepileptic drug treatment. A high-dose antiepileptic drug treatment refers to one of the following situations: valproic acid sodium more than 800mg per day, lamotrigine more than 150mg per day, carbamazepine more than 500mg per day, oxcarbazepine more than 1000mg per day, phenytoin more than 250mg per day, or antiepileptic drug treatment with more than three different drugs at the same time.
